# Safety, Fear and Neuromuscular Responses after a Resisted Knee Extension Performed to Failure in Patients with Severe Haemophilia

**DOI:** 10.3390/jcm10122587

**Published:** 2021-06-11

**Authors:** Joaquín Calatayud, Jonathan Martín-Cuesta, Juan J. Carrasco, Sofía Pérez-Alenda, Carlos Cruz-Montecinos, Lars L. Andersen, Felipe Querol-Giner, José Casaña

**Affiliations:** 1 Exercise Intervention for Health Research Group (EXINH-RG), Department of Physiotherapy, University of Valencia, 46010 Valencia, Spain; joaquin.calatayud@uv.es (J.C.); jonimartin_2_@hotmail.es (J.M.-C.); jose.casana@uv.es (J.C.); 2 National Research Centre for the Working Environment, 2100 Copenhagen, Denmark; lla@nfa.dk; 3 Physiotherapy in Motion Multispeciality Research Group (PTinMOTION), Department of Physiotherapy, University of Valencia, 46010 Valencia, Spain; sofia.perez-alenda@uv.es (S.P.-A.); carcruz2@alumni.uv.es (C.C.-M.); felipe.querol-giner@uv.es (F.Q.-G.); 4 Intelligent Data Analysis Laboratory, University of Valencia, 46010 Valencia, Spain; 5 Haemostasis and Thrombosis Unit, Universitary and Polytechnic Hospital La Fe, 46010 Valencia, Spain; 6 Laboratory of Clinical Biomechanics, Department of Physical Therapy, Faculty of Medicine, University of Chile, Santiago 8380453, Chile; 7 Sport Sciences, Department of Health Science and Technology, Aalborg University, 9220 Aalborg, Denmark

**Keywords:** EMG, neuromuscular fatigue, quadriceps, arthropathy, resistance training, kinesiophobia

## Abstract

Background: low–moderate intensity strength training to failure increases strength and muscle hypertrophy in healthy people. However, no study assessed the safety and neuromuscular response of training to failure in people with severe haemophilia (PWH). The purpose of the study was to analyse neuromuscular responses, fear of movement, and possible adverse effects in PWH, after knee extensions to failure. Methods: twelve severe PWH in prophylactic treatment performed knee extensions until failure at an intensity of five on the Borg CR10 scale. Normalised values of amplitude (nRMS) and neuromuscular fatigue were determined using surface electromyography for the rectus femoris, vastus medialis, and vastus lateralis. After the exercise, participants were asked about their perceived change in fear of movement, and to report any possible adverse effects. Results: Patients reported no adverse effects or increased fear. The nRMS was maximal for all the muscles before failure, the median frequency decreased, and wavelet index increased during the repetitions. The vastus lateralis demonstrated a higher maximum nRMS threshold and earlier fatigue, albeit with a lower and more progressive overall fatigue. Conclusions: severe PWH with adequate prophylactic treatment can perform knee extensions to task failure using a moderate intensity, without increasing fear of movement, or adverse effects.

## 1. Introduction

Haemophilia is a rare bleeding disorder related to the X chromosome which is caused by the deficiency of clotting factors VIII (haemophilia A) and IX (haemophilia B) [1]. Approximately 40% of people with haemophilia (PWH) A and 30% of PWH B present plasma coagulation factor levels below 1% and therefore suffer from severe haemophilia [2]. A lack of clotting factors makes patients prone to abnormal bleeding, with 80% of these bleeds occurring in the musculoskeletal system in the form of hemarthrosis and intramuscular bruising [3]. 

Repeated hemarthrosis causes changes in the structure of the synovial membrane and can evolve into chronic synovitis, which can result in joint degeneration, known as haemophilic arthropathy [3]. The knee is one of the most affected joints [4], with haemophilic arthropathy being associated with pain [5], secondary soft tissue contractures, muscular atrophy, angular deformities, and loss of range of motion [6], thus limiting the quality of life of PWH. The gold standard care to prevent all the aforementioned consequences is regular replacement therapy (prophylactic treatment) with the deficient clotting factor [7]. In addition to prophylactic treatment, strength training has been postulated as a valid therapeutic option for the management and prevention of repetitive hemarthrosis and for the improvement of muscle function [8]. Controlled strength training is appropriate for PWH because, up to a certain intensity, it appears to be safe; does not involve impacts or falls [9]; may decrease the frequency of pain and bleeding; and improves strength and hypertrophy, which is closely related to the ability of patients to perform the activities of daily living [10], proprioception, and bone mineral density [11]. 

The American College of Sports Medicine [12] recommends, for healthy people, intensities of 70–85% of one maximum repetition (1RM) to generate hypertrophy. However, recent studies have shown that low-moderate intensity training, in the 40–80% 1RM range, can also effectively increase strength and muscle hypertrophy in healthy people with no previous experience when they train to task failure [13]. Indeed, regardless of the intensity or duration of the repetitions, training to task failure exhausts glycogen deposits resulting in anabolic signaling in Type I and II muscle fibres, which usually occurs prior to their activation [14]. According to the size principle [15], training until task failure could promote higher electromyographic activity, suggesting the recruitment of high-threshold motor units, which could translate into better hypertrophic adaptations [16]. In contrast, other studies suggest that these adaptations can be achieved some repetitions before this point [17,18]. For example, in healthy untrained women, a complete activation of the deltoids and shoulder and neck stabilising muscles was found during lateral shoulder lifts with a 15RM load three to five repetitions before reaching task failure [18]. Importantly, training to failure could generate high levels of discomfort or pain [19], likely decreasing exercise adherence. Another potential problem of carrying out exercises until failure is the patient’s possible fear of movement (i.e., kinesiophobia), because many PWH have been avoiding exercise as a result of a fear of causing bleeds. Nonetheless, to date, no studies have assessed the safety and neuromuscular response of training to failure in these patients.

The objectives of this study were to analyse neuromuscular responses, fear of movement, and possible adverse effects in patients with severe haemophilia, after performing a resisted knee extension exercise to failure. We hypothesised that patients would be able to perform a moderate intensity strength exercise until failure without increased fear of movement or adverse effects (pain or bleedings). We also posited that, with a progressive increase in neuromuscular fatigue, PWH would reach a maximum muscle activity amplitude before reaching failure.

## 2. Materials and Methods

### 2.1. Participants

Patients aged over 18 years, diagnosed with severe haemophilia in prophylactic treatment were considered as candidates for the present study and were asked to participate. Patients were excluded if they had undergone a joint replacement in the year prior, had experienced any joint or muscle bleeding in the past three months, or if they had a medical condition which contraindicated engaging in exercise. A total of 12 patients with severe Type A haemophilia voluntarily participated in this study, which was carried out at the University of Valencia (Spain) during March 2020. All the participants were informed in advance about the purpose and content of the research and written informed consent was obtained from all of them. This work was carried out in accordance with the Declaration of Helsinki and approved by the Ethics Committee (H1461147538087). The data we collected formed part of a larger research project on muscle activation and the tolerance and effort perception in haemophilia patients while performing knee extension exercises with different elastic bands.

### 2.2. Procedures

The following clinical variables were collected: type of haemophilia (A or B) and coagulation factor dose (International Units/Kg) before the experimental session.

Each participant carried out one experimental session and were asked to abstain from consuming food, drinks, or stimulants such as caffeine 2 h before the session, and not to do any physical activity more intense than their normal daily activities for 24 h before. In addition, they were instructed to sleep at least 7–8 h the night before the data collection session. All the measurements were recorded by the same researchers and were carried out in the same University facility. Participants performed the experimental session 1 to 2 h after receiving their usual prophylactic clotting factor treatment.

During the experimental session, the patients’ height (IP0955, Invicta Plastics Limited, Leicester, England), and weight (BF-350; Tanita, Tokyo, Japan), were measured. The Hemophilia Joint Health Score 2.1 (HJHS) was then used to assess joint health. The HJHS provides joint-specific scores ranging from 0 to 20, with a total score from 0 to 124, where higher scores reflect poorer condition [20]. 

Next, the Tampa Scale for Kinesiophobia (TSK-11) [21] was used to assess the initial patient fear of movement. This questionnaire was chosen because PWH may have an increased fear of movement derived from the possibility that exercise could cause episodes of bleeding or repeated injury. Subsequently, the participants answered a brief questionnaire about the physical activity they engaged in during their free time [22] and their experience with strength training. Next, they completed two scales that rated fear from zero to ten, with zero being no fear and ten being the maximum possible fear, in relation to performing strength exercise and to completing this exercise to the point of task failure. 

Finally, the surface electromyography (EMG) measurement protocol was used to measure the maximum voluntary isometric contraction (MVIC) during exercise. We prepared each patient’s skin by shaving the hair on the muscles of interest and cleaning the skin with a cotton swab dipped in alcohol before placing the electrodes, following the SENIAM (surface electromyography for the non-invasive assessment of muscles) recommendations [23] for the rectus femoris (RF), vastus lateralis (VL), and vastus medialis (VM) of the quadriceps on the dominant side of the lower body. Specifically, we used disk-shaped silver/silver chloride (Ag/AgCl) bipolar 44.8 × 22 mm electrodes (Sensor Blue N-00-S; Ambu A/S, Ballerup, Denmark) with a measurement area of 95 mm^2^, located at a distance of 2 cm. The reference electrode was placed a finger’s distance for the RF and on the patella for the VL and VM, according to the manufacturer’s specifications. EMG data was assessed by using Shimmer3 sensors (Shimmer Sensing, Dublin, Ireland), with the signal processed through “The mDurance system” software (mDurance solutions, Granada, Spain).

All the signals were collected at a sampling frequency of 1024 Hz with a bandwidth of 8 to 500 Hz. The common mode rejection ratio was 110 dB. Before the patient started the exercise, we checked the offset values for each channel to make sure they were within the ± 2 μV range of 0 μV. All the myoelectric activity records (in microvolts) were stored on a hard disk for subsequent analysis. Before completing the exercise described below, we measured two 5-s MVICs with a 1-min rest interval. The participants also performed a non-maximal test to make sure they understood the task. They were then asked to exercise a progressive contraction for 2 s with 3 s of maximum contraction. Verbal encouragement was provided to motivate all participants to reach their maximal effort.

Positioning during the MVIC was based on standardised muscle testing procedures for the RF, VL, and VM [24], and a knee extension exercise was used. This was performed in a sitting position with 70° of knee flexion and 110° of hip flexion, performed against a fixed resistance for the RF and maintaining a neutral ankle dorsiflexion at 90°. After 2 min of rest, the participants performed 8 light intensity repetitions to warm up, followed by 7 additional sets of three repetitions with 2 min of rest between each set until they found the elastic band that required an effort of five, which corresponds to a moderate intensity on the Borg CR10 scale [25]. 

Participants were provided seven elastic bands (TheraBand CLX, The Hygenic Corporation, Akron, OH, USA) with different resistances (yellow, red, green, blue, black, silver, and gold). After finding the elastic band that corresponded to five on the Borg CR10 scale, the patient completed the exercise test, with verbal encouragement, until task failure. The test was carried out with same position used for the MVICs. In addition, the participants were asked to perform the exercise using the greatest possible range of motion they could, as closely as possible to the original technique they had been shown. The patients were asked not to use their trunk, as far as possible, but were allowed to hold onto the sides of the bench with their hands. In order to perform the exercise without sudden jerks or accelerations, a metronome was used to standardise the speed of movement to 1.5 s each for the concentric and eccentric phases, and feedback was provided if we noted any deviation by any participant. 

At the end of the exercise, we noted the number of repetitions they had performed and their pain levels, assessed by the 11-point numerical pain scale. Moreover, the participants were asked to complete two global change scales about fear of doing strength exercises generally or completing these exercises to task failure, with seven possible answers: very much improved, much improved, minimally improved, no change, minimally worse, much worse, very much worse. Finally, 24 and 48 h after the session, the participants were asked about any possible adverse effects they had experienced (bleeding, pain, etc.) and they were also asked to report any possible adverse effects during the following week.

### 2.3. Data Analysis

The EMG signals were processed using algorithms developed with MATLAB (The MathWorks Inc., Natick, MA, USA, version R2015a). The signals acquired during the exercises were digitally filtered using a 10Hz high pass filter. The following indices were then obtained:

(1) Maximum repetition values. A moving root-mean-squared (RMS) smoothing filter was applied to the EMG signals, implemented with a 500ms window (250ms backward and 250ms forward) for each signal sample. The peaks of maximum and minimum values of the RMS corresponding to each of the contractions performed were obtained for all three muscles. The maximum RMS activation percentage (amplitude) was calculated by normalising the result of each condition and muscle to the highest RMS activation value reached by the participant during the whole experimental session (nRMS), including MVICs, so a true maximum value was obtained.
(2) Median frequency (MF). The original EMG signal was segmented into individual contractions based on the maximum and minimum values of the RMS EMG. To establish the start and end of each contraction, we considered a minimum activation of 10% with respect to the difference between the maximum and minimum values of each contraction. The MF was obtained from the power spectrum of each of the contractions registered in the EMG by applying a Fourier transformation. The MF represents the frequency at which the power spectrum of a signal was divided into two equal halves. The final MF value was normalised to the maximum MF value in the series.
(3) The wavelet index between wavelengths at different scales (*WIRW*51) [26]. This index was more adequate to assess changes in muscle output than the traditional MF and other approaches, even in the presence of additive Gaussian noise in the EMG signal [26,27]. The discrete wavelet transform (DWT) breaks a signal into successive approximations using a wavelet function ψ (*t*) and a scaling function ϕ (*t*). The *WIRW*51 index expresses the ratio of the signal amplitude changes occurring at two different scales. Its mathematical expression is as follows:
  (1) $$WIRW51=\frac{\sum_{i=2}^{N}{\left|D_{5}\left(i\right)-D_{5}\left(i-1\right)\right|}^{2}}{\sum_{i=2}^{N}{\left|D_{1}\left(i\right)-D_{1}\left(i-1\right)\right|}^{2}}$$
  where *D*_5_(n) and *D*_1_(n) are the signal approximations in scales five and one, obtained by DWT with the sym5 wavelet function. The value of the *WIRW*51 index was also normalised to the maximum value of the session for each participant. Finally, the three parameters analysed were normalised in intervals of 20% of the total number of repetitions until task failure was reached. Neuromuscular fatigue was determined by a significant decrease in MF and significant increase in nRMS [28] and *WIRW*51 [26,27] over time. Since these parameters do not necessarily change simultaneously, all of them were needed to consider the occurrence of neuromuscular fatigue.

### 2.4. Statistical Analysis

The statistical analysis was performed with the SPSS software (Version 26; IBM Corp, Armonk, NY, USA). The normality of the data was verified with the Shapiro–Wilk test. Descriptive results are shown as means and standard deviations. To analyse the parameters obtained in the different repetition intervals, repeated measures analysis of variance (ANOVA) models were used. When the main effects indicated significant differences, the Bonferroni correction was applied to avoid Type I error caused by multiple comparisons. Effect size was interpreted as small (d = 0.2), medium (d = 0.5) and large (d > 0.8). The data were considered to be statistically significant when *p* < 0.05. 

An a priori power analysis was conducted with G∗Power (version 3.1.9.2; Heinrich-Heine-Universität Düsseldorf, Düsseldorf, Germany) software to calculate the sample size. With the present study design, assuming a medium effect size (f = 0.30), an alpha value of 0.05, a power of 0.80, and a correlation among repeated measures of 0.6, the total sample size required is 12 participants.

## 3. Results

### 3.1. Descriptive, Safety and Fear of Movement Data

Twelve participants with a mean age of 38.4 (10.1) years were included in this study. Demographic and descriptive data are shown in Table 1. 

Only two patients showed initial knee pain, with intensity values of four and one, respectively. No adverse effects or pain were found 24 h after the experimental session. Regarding the improvement in fear of strength training as well as in fear of performing exercises until failure, after completing the experimental session, two subjects stated that their levels of fear had “very much improved”, four said it had “much improved”, one said it had “minimally improved”, and five reported “no change”. Table 2 shows baseline kinesiophobia levels and Table 3 shows individual data for the task to failure.

### 3.2. Normalised Values of Electromyographic Amplitude

**Figure 1:**
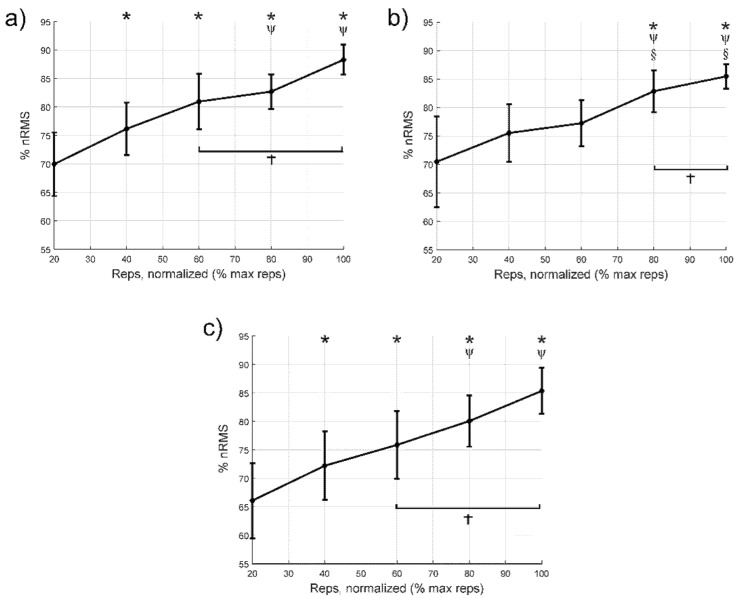
Variations in the normalised values of electromyographic amplitude (nRMS) during each cycle from 20% to failure; (**a**) vastus medialis, (**b**) vastus lateralis, and (**c**) rectus femoris. * Indicates statistically significant differences (*p* < 0.05) compared to the first 20% cycle. Ψ Indicates significant differences compared to the 40% cycle. § Indicates significant differences compared to the 60% cycle. † Indicates cycles with no significant difference from the 100% cycle. The diamonds indicate the mean value, while the bars express the 95% confidence interval of the mean. nRMS: normalised values of electromyographic amplitude.

Figure 1 shows the relationship between the repetitions performed and the nRMS. In the VM muscle (Figure 1a), no statistically significant differences were found between 100% and 60% (*p* = 0.16) of cycle or between 100% and 80% (*p* = 0.29). In the VL (Figure 1b), no statistically significant differences were found between 100% and 80% (*p* = 0.62). Finally, in the RF (Figure 1c), no statistically significant differences were found between 100% and 60% (*p* = 0.09) or 100% and 80% (*p* = 0.16).

### 3.3. Median Frequency

**Figure 2:**
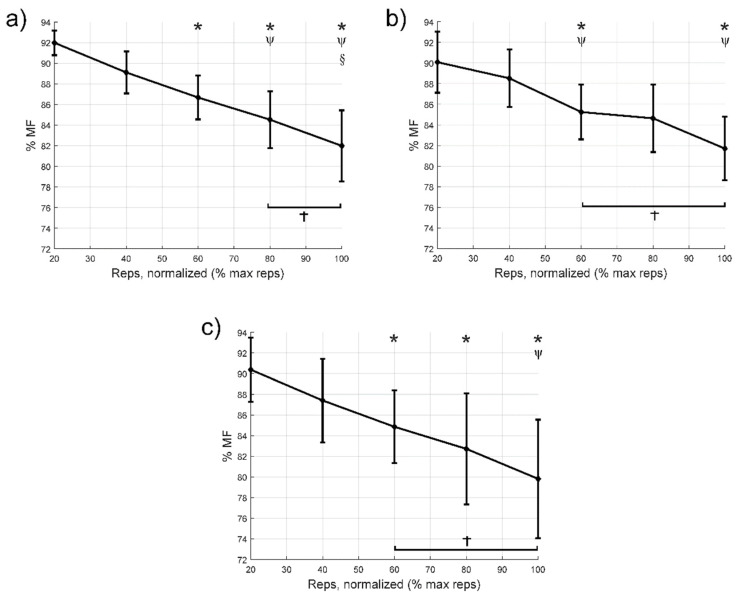
Variations in the median frequency during each cycle from 20% to failure; (**a**) vastus medialis, (**b**) vastus lateralis, and (**c**) rectus femoris. * Indicates statistically significant differences (*p* < 0.05) compared to the first 20% cycle. Ψ Indicates significant differences compared to the 40% cycle. § Indicates significant differences compared to the 60% cycle. † Indicates cycles with no significant difference from the 100% cycle. Diamonds indicate the mean value, while bars express the 95% confidence interval of the mean. MF: median frequency.

Figure 2 shows the neuromuscular fatigue expressed through the MFs during the different repetitions. In the VM muscle (Figure 2a), no statistically significant differences were found between 100% and 80% of cycle (*p* = 0.11). In the LV (Figure 2b), no statistically significant differences were found between 100% and 60% (*p* = 0.36) or 100% and 80% (*p* = 0.27). Lastly, in the RF muscle (Figure 2c), no statistically significant differences were found between cycles 100% and 60% (*p* = 0.12) or 100% and 80% (*p* = 0.14).

### 3.4. Wavelet Index between Wavelengths at Different Scales

**Figure 3:**
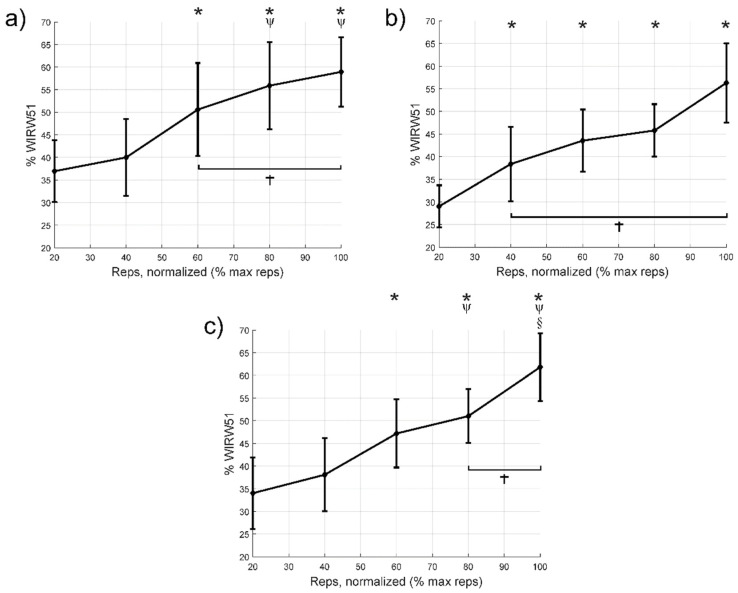
Variations in the *WIRW*51 wavelet index during each cycle, from 20% to failure; (**a**) vastus medialis, (**b**) vastus lateralis, and (**c**) rectus femoris. * Indicates statistically significant differences (*p* < 0.05) compared to the first 20% cycle. Ψ Indicates significant differences compared to the 40% cycle. § Indicates significant differences compared to the 60% cycle. † Indicates cycles with no significant difference from the 100% cycle. Diamonds indicate the mean value, while bars express the 95% confidence interval of the mean.

Figure 3 shows the changes in *WIRW*51 in the VM, VL, and RF throughout the repeats. In the VM muscle (Figure 3a), no statistically significant differences were found between cycles 100% and 60% (*p* = 0.59) or 100% and 80% (*p* = 1.0). In the VL muscle (Figure 3b), no statistically significant differences were found between cycles 100% and 40% (*p* = 0.15), 100% and 60% (*p* = 0.14), or 100% and 80% (*p* = 0.05). Finally, in the RF muscle (Figure 3c), no statistically significant differences were found between cycles 100% and 80% (*p* = 0.14).

Table 4 shows the differences in means (95% confidence intervals), *p* values and effect size for comparisons between the first 20% cycle and the remaining intervals until failure for the nRMS, MF and *WIRW*51 variables. Compared to the first 20% cycle, there was an increase in nRMS from the 40% cycle, except in the VL, where the first increase occurred from the 80% cycle onwards. Regarding MF, there was a decrease from the 60% cycle onwards. Finally, the *WIRW*51 index increased from the 60% cycle, except for the VL, which increased from the 40% of cycle. In the cases with significant differences, the effect size was medium or large.

## 4. Discussion

The main findings of the study were that severe PWH who receive adequate prophylactic treatment can perform a knee extension to task failure using a moderate intensity, without increasing pain, fear of movement, or adverse effects. Importantly, going to complete failure is not necessary to elicit a maximal nRMS response. Specifically, there was maximal nRMS of the VL, VM, and RF at 60–80% of the total number of repetitions, and an increase in neuromuscular fatigue throughout the repetitions during the knee extension with moderate resistance. We observed an increase in nRMS during the repetitions, suggesting an increase in overall muscle fibre recruitment for a fixed load [29,30] as well as an increase in firing rate and better motor unit recruitment timing or impaired excitation–contraction coupling [30]. This was accompanied by a decrease in MF and an increase in *WIRW*51. Increased recruitment of high-threshold motor units, following the Henneman principle, and synchronisation between motor units are also factors that likely explain part of the increase in nRMS and decrease in the MF [14,15]. Furthermore, increased concentration of the lactates when fatigue raises, decreases intracellular pH, reducing muscle fibre conduction velocity and in turn, MF [31]. A decrease in muscle fibre conduction velocity is also one of the causes explaining the increase in nRMS because of a spatial low-pass filtering effect of tissue as a volume conductor [32]. Overall, our results suggest that terminating the each set a few repetitions short of failure could be sufficient to stimuli muscle strength and hypertrophy.

In the present study, the VL reached its maximum nRMS at 80% of the total number of repetitions, which is equivalent to around seven repetitions before reaching task failure. Similarly, a previous study found that the myoelectric activity of the deltoid and shoulder and neck stabilising muscles in untrained women during lateral shoulder lifts was maximum at three to five repetitions before task failure [18]. However, differences in the type of patients must also be taken into account, because patients with advanced knee deterioration have shown neuromuscular control dysfunction in the quadriceps [33]. Another aspect worth mentioning is the difference between studies in terms of the number of repetitions used and their intensity. However, a recent study [14] found that the depletion of glycogen deposits, which indicates a previous activation of muscle fibres, and anabolic signalling in both Type I and II fibres, was independent of the intensity and duration of exercise when the task was taken to failure. Furthermore, the high number of repetitions reached by some patients in our study could be explained if they had overestimated their perceived effort during the test [34], a decision which may be influenced by a fear of using very high intensities that could generate discomfort or adverse effects.

In our study, the VM and RF reached their maximum nRMS in the third to last cycle (60% of the total number of repetitions), at around 15 repetitions before reaching task failure. Similarly, a recent study found a progressive VM myoelectric activity increase during a knee extension exercise at 10RM in patients with total knee arthroplasty, with maximal activation occurring at approximately 70% of the total number of repetitions [35]. Neuromuscular fatigue in the VL, VM, and RF, interpreted based on the MF, also decreased from start to finish, with this change being significant after 60% of the repetitions. Jenkins et al. [36] also found a progressive decrease in the MF when healthy participants performed knee extensions up to task failure at intensities of 30% and 80% of 1RM. However, they observed a more marked decrease when exercising at 30% of 1RM, suggesting that the mechanisms of neuromuscular fatigue that led to task failure differ according to the specific exercise intensity.

In contrast, it should be noted that the behaviour of the VL with respect to the VM and RF differed in some respects in this present study. For the VL, there were differences in the MF between the first cycle and 60% of the repetitions, but not compared to 80% of them. Furthermore, using the *WIRW*51, we observed that neuromuscular fatigue appeared first in the VL, although it remained more stable during the remaining repetitions and did not reach values as high as in the VM and RF. However, previous studies have reported contrasting results. For example, a study that performed knee extensions at 50% of 1RM until failure in healthy and active subjects verified that the decrease in MF was lower for the VM compared to the VL and the RF, regardless of sex [37]. Another previous study by Grabiner et al. [38] found no difference in the quadriceps muscle MF in healthy men while performing isometric or isotonic knee extensions. Despite this and considering the increased sensitivity of the *WIRW*51 compared to MF to detect fatigue in the quadriceps during dynamic contractions [26,27], we believe that our results are logical and further complement the more attenuated MF response and higher nRMS threshold we found in the VL.

The differences in nRMS and neuromuscular fatigue that we found in the VL with respect to the VM and RF could have several explanations, including differences in their anatomical structure [39,40], biomechanical function [41], and morphology [42,43]. Interestingly, a study [44] found a relationship between the number of neuromuscular spindles, the firing rate, and muscle recruitment in isometric contractions. Specifically, muscles with fewer spindles exhibited higher firing rates and a lower recruitment threshold, while muscles with more spindles received greater negative feedback, which reduced their firing rates, increased their maximum recruitment threshold, and changed the distribution of motor neuron recruitment. These data could justify the differences in the timing of the maximum nRMS and in the pattern of neuromuscular fatigue we observed between the VL (which has a greater number of spindles) and the VM and RF (which contain fewer spindles) [44], and a lower recruitment threshold meaning that they reach the point of maximum nRMS earlier.

The findings of the present study show that training until task failure is not necessary for maximal quadriceps activation in severe PWH, and that they reach a significant level of neuromuscular fatigue at moderate intensities. This data can be used at a practical level so that strength programs could improve hypertrophy and strength without having to achieve the unpleasant feeling of reaching task failure. More importantly, these improvements could be achieved safely, without increased pain or adverse effects, and without increasing fear of strength training and reaching task failure. In fact, our study demonstrated that most of the participants improved their fear of movement after performing the task to failure. This is most likely because of the lack of training experience to task failure among most of the participants, meaning that self-efficacy improved after they had completed the set without any difficulties. This is very important in severe PWH because fear of movement can mean that these individuals get into a vicious circle where muscle disuse increases [45], thus increasing their risk of muscle atrophy and joint instability, pushing them towards states of chronic pain, an increased chance of bleeding [46], and a decreased quality of life. Unfortunately, little is known on how the perceptions of discomfort, pain, and kinesiophobia can interfere on the neuromuscular fatigue in PWH and the adherence of strength training. Nonetheless, the prophylactic treatment may need to be mandatory to perform heavy exercise training, especially in those with severe and moderate haemophilia. Furthermore, the delayed onset of muscle soreness may also be a factor that can influence adherence and performance during routine prescription. Future studies are needed to better understand how symptoms and adherence is affected by exercise with and without training to task failure.

The current study has limitations, including its small sample size. However, according to a previous power analysis the number of participants was sufficient for this design. Some limitations inherent to EMG use must be considered, since global EMG estimate, such as the surface EMG amplitude and power spectral frequencies are generally largely variable among subjects and do not reflect motor unit recruitment [47]. In addition, the processing and interpretation of surface interference EMG signals, in particular, using signal amplitude, is challenging and should be done with caution. Our results could not be replicated in PWH with greater arthropathy levels. Our measurements were conducted in the dominant side, which in general had greater joint health (as can be observed at the HJHS scores). However, evaluating the dominant side is a generally conducted practice in similar EMG studies (specially to avoid additional fatigue) and we were not interested in comparing results between more vs. less damaged joints. Moreover, since we evaluated for the first time a task to failure with a moderate intensity in these patients (which translates into a high number of reps), we considered that this would be a better approach. It should be highlighted that while the maximum nRMS in the quadriceps can be achieved without the need to reach task failure, the present study does not demonstrate that this training would improve muscle strength and hypertrophy in the same extent as training to task failure in PWH. The study presented here does lay the physiological groundwork for the safe performance of future experimental studies potentially to demonstrate this hypothesis. Finally, the participants were only asked to report adverse events, and so it is possible that subclinical bleeding or minor adverse events may have been overlooked.

## 5. Conclusions

Severe PWH who receive adequate prophylactic treatment can perform a knee extension to task failure using a moderate intensity, without increasing fear of movement, or adverse effects (bleeding, pain). However, the maximum nRMS in the quadriceps can be achieved without the need to reach failure. Neuromuscular fatigue presents a different pattern for each muscle, with the VL demonstrating a higher maximum nRMS threshold and earlier fatigue, albeit with a lower and more progressive overall fatigue.

## Figures and Tables

**Table 1:** Demographic and descriptive data.

(*n* = 12)	Mean (SD)
Age (years)	38.4 (10.1)
Height (cm)	174.3 (9.0)
Body mass (kg)	78.0 (11.3)
HJHS ^1^ dominant knee	0.7 (1.1)
HJHS ^1^ non-dominant knee	3.4 (6.0)
Total HJHS ^1^	25.6 (13.3)
FVIII dose (IU/Kg) ^2^	25.0 (10.3)
	No of patients
Knee pain intensity (0/1/2/7) ^3^	9/1/1/1
**Leisure-Time Physical Activity**	
**Frequency**	***n* (%)**
Never	0 (0.0)
<1 time/week	1 (8.3)
1 time/week	1 (8.3)
2–3 times/week	5 (41.7)
Almost daily	5 (41.7)
Intensity	
Relatively easy	7 (58.3)
Fairly strenuous	4 (33.3)
Near the limit of exhaustion	1 (8.3)
Duration	
<15 min	
16–30 min	1 (8.3)
30–60 min	5 (41.7)
>1 h	6 (50.0)
Resistance training	
Yes	8 (66.7)
No	4 (33.3)
Frequency	
1 time/week	2 (25.0)
2 times/week	4 (50.0)
3 times/week	1 (12.5)
4 times/week	1 (12.5)
Years of experience	
1 year	2 (25.0)
2 years	2 (25.0)
≥3 years	4 (50.0)
Intensity	
Moderate (60–70%)	7 (87.5)
Heavy (>80%)	1 (12.5)

^1^ HJHS; hemophilia joint health score. ^2^ Coagulation factor dose before the experimental session. ^3^ 11-point numerical pain scale.

**Table 2:** Baseline kinesiophobia.

Item Number	Mean (SD)
1	2.5 (1.1)
2	2.9 (1.1)
3	1.9 (0.9)
4	2.5 (1.3)
5	2.6 (1.4)
6	2.6 (1.1)
7	2.7 (1.0)
8	3.4 (0.8)
9	1.6 (0.9)
10	2.7 (0.9)
11	2.3 (1.3)
Total	27.7 (7.0)

**Table 3:** Individual data for the task to failure.

Patient	Selected Resistance Band	No of Repetitions	Pain (Post)	Fear of Exercise to Task Failure Pre (0–10)	Fear Improvement (Post)
1	Silver	35	4	5	Has improved something
2	Silver	59	0	3	Has improved a lot
3	Silver	60	0	2	Has not changed
4	Silver	34	0	5	Has improved something
5	Gold	30	0	0	Has not changed
6	Black	31	0	2	Has not changed
7	Gold	14	0	0	Has improved something
8	Silver	65	2	5	Has not changed
9	Gold	64	0	3	Has improved something
10	Gold	27	0	5	Has improved something
11	Gold	36	0	6	Has improved a lot
12	Silver	82	0	1	Has minimally improved

**Table 4:** Mean differences [95% confidence intervals], *p* values and effect size for comparisons from the first 20% cycle to the remaining intervals until failure for nRMS, MF and *WIRW*51.

		Vastus Medialis	Vastus Lateralis	Rectus Femoris
		nRMS		
Cycle to failure (%)	40	−6.2 [−11.5:−0.9]; **0.019; 0.77**	−5.1 [−12.1:2.0]; 0.29;	−6:1 [−11.6:−0.7]; **0.024; 0.63**
	60	−11.0 [−16.6:−5.4]; **<0.001; 1.33**	−6.8 [−15.5:1.9]; 0.20;	−9.8 [−19.1:−0.5]; **0.035; 1.01**
	80	−12.8 [−19.9:−5.6]; **<0.001; 1.80**	−12.4 [−22.1:−2.7]; **0.009; 1.28**	−14.0 [−21.5:−6.5]; **<0.001; 1.61**
	100	−18.3 [−28.8:−7.9]; **<0.001; 2.68**	−15.0 [−27.4:−2.7]; **0.014; 1.64**	−19.3 [−30.5:−8.1]; **<0.001; 2.30**
		MF		
Cycle to failure (%)	40	2.9 [−0.3:6.0]; 0.086;	1.6 [−2.7:5.9]; 1;	3.0 [−0.2:6.2]; 0.08;
	60	5.3 [1.3:9.3]; **0.007; 1.96**	4.8 [1.0:8.7]; **0.011; 1.09**	5.5 [1.7:9.3]; **0.003; 1.06**
	80	7.5 [2.0:12.9]; **0.006; 2.24**	5.5 [−0.8:11.7]; 0.11;	7.7 [0.7:14.6]; **0.026; 1.11**
	100	10.0 [3.8:16.3]; **0.002; 2.46**	8.3 [2.0:14.8]; **0.008; 1.76**	10.6 [2.5:18.7]; **0.008; 1.46**
		*WIRW*51		
Cycle to failure (%)	40	−3.1 [−12.7:6.6]; 1;	−9.4 [−17.7:−1.0]; **0.024; 0.89**	−4.1 [−13.8:5.6]; 1;
	60	−13.7 [−24.4:−2.9]; **0.010; 0.99**	−14.5 [−22.8:−6.2]; **<0.001; 1.57**	−13.2 [−23.6:−2.7]; **0.011; 1.09**
	80	−19.0 [−28.2:−9.7]; **<0.001; 1.44**	−16.8 [−28.3:−5.2]; **0.004; 2.02**	−17.0 [−25.9:−8.1]; **<0.001; 1.54**
	100	−22.0 [−33.5:−10.5]; **<0.001; 1.93**	−27.3 [−44.2:−10.4]; **<0.001; 2.47**	−27.8 [−44.9:−10.7]; **<0.001; 2.30**

Significant differences are shown in bold.

## Data Availability

The data underlying this article will be shared on reasonable request to the corresponding author.
